# Supplementary material for: Intracontinental deformation of the Tianshan Orogen in response to India-Asia collision
Source: Nat Commun. 2022 Jun 29;13:3738. doi: 10.1038/s41467-022-30795-6 (PMC9243081; doi:10.1038/s41467-022-30795-6)
Supplement: Supplementary file 1 — Supplementary Information [file 41467_2022_30795_MOESM1_ESM.pdf]

Supplementary Information for

Intracontinental deformation of the Tianshan Orogen in response to India-Asia collision

Wei Li[1, 2, 3], Yun Chen[1, 2, *], Xiaohui Yuan[3], Wenjiao Xiao[4, 1, 5, *], Brian F. Windley[6]

[1]State Key Laboratory of Lithospheric Evolution, Institute of Geology and Geophysics, Chinese Academy of Sciences, Beijing 100029, China
[2]CAS Center for Excellence in Deep Earth Science, Guangzhou, 510640, China
[3]Deutsches GeoForschungsZentrum GFZ, Potsdam 14473, Germany
[4]Xinjiang Research Center for Mineral Resources, Xinjiang Institute of Ecology and Geography, Chinese Academy of Sciences, Urumqi 830011, China
[5]College of Earth and Planetary Sciences, University of Chinese Academy of Sciences, Beijing 100049, China
[6]School of Geography, Geology and the Environment, University of Leicester, Leicester LE1 7RH, UK
[*]Corresponding author, E-mail: yunchen@mail.iggcas.ac.cn; wj-xiao@mail.iggcas.ac.cn

Contents

Supplementary Text 1: Comparison of our results and previous studies.

Supplementary Figure 1: Examples for the quality controls and harmonic analysis of P-wave receiver functions (RFs) with Gaussian coefficients 1.0.

Supplementary Figure 2: Average RFs, interpolated Rayleigh wave dispersions (DPs), and inverted 1-D S-wave velocity (V_S) model at each station.

Supplementary Figure 3: Checkerboard tests and posterior errors of group and phase velocity DPs.

Supplementary Figure 4: Examples of the joint inversion of RFs and DPs.

Supplementary Figure 5: Robustness tests of crustal low-V_S anomalies and mantle lid structure.

Supplementary Figure 6: CCP stacking results using the *Pms* phase of the reliable RFs.

Supplementary Figure 7: CCP stacking results using the *PpPs* phase of the reliable RFs.

Supplementary Figure 8: Robustness tests of CCP stacking with different smoothing schemes.

Supplementary Figure 9: Robustness tests of CCP stacking with different data amount.

Supplementary Figure 10: Synthetic tests for effects of shallow structures in the CCP stacking.

Supplementary Figure 11: Comparison of our result with previous studies.

Supplementary References

Supplementary Text 1. Comparison of our results and previous studies

The Moho geometry revealed in this study corresponds well to the tectonic terranes across the Tianshan, i.e., Issyk Kul Arc (IAK), Naryn Arc (NA), and South Tianshan Accretionary Complex (STAC) (Fig. 2c). The NA has a nearly flat Moho with the depth of ~45 km similar to that beneath the Tarim Craton and the Kazakh Shield. The Moho gradually dips to the south near the Atbashy-Inylchek Fault (AIF) and reaches its deepest level of ~70 km beneath the STAC, in contrast to the Moho doublet that was revealed beneath the NL in which the Moho depth changed abruptly from ~45 km beneath the NA to ~60 km beneath the IKA. Beneath the Tarim Craton and the Kazakh Shield, intracrustal positive amplitudes emerge at the depth of 20–25 km along with the Vs increasing to ~3.7 km/s (Fig. 2d) and likely represent the Conrad discontinuity that is considered to be the interface between the upper and lower continental crust. These positive intracrustal *Ps* phases are so weak beneath the NA and the IKA that they can only be identified in the Vs model and they totally disappear beneath the STAC, which has an extensive low-V_S middle-lower crust (Fig. 2d). Another prominent intracrustal feature concerns the negative *Ps* phases beneath the NA and IKA that are coincident with the banded low-V_S anomalies in our V_S model (Fig. 2d).

Similar Moho and intracrustal interfaces across the Tianshan were also demonstrated by previous studies[1,2,3]. More detailed geometrical characteristics can be identified when combining our new constructed images with previous results, and these provide a more reliable and unified crustal model related to the Tianshan's architecture and deformation. As shown in Supplementary Fig. 11, the lower crust of the Tarim Craton is featured with significant increase of the V_S beneath the flat intracustal positive signals (IP1) at a depth of ~25 km, indicating that the shallow intracrustal positive signals (IP2) observed in the Tarim Craton depicts upper crustal detachments that have intense seismic activity. Therefore, the northward extension of the high-V_S lower crust and the shallow seismic activity suggest that the underthrusting of the Tarim Craton should end near the North Tarim Fault (NTF). Although another northward-extending intracrustal positive signal (IP3) beneath the STAC[2] cannot be detected in our CCP images, it may exist in our Vs model where it separates the low-V_S crust of the STAC from the Tarim Craton (Supplementary Fig. 11). Southward underthrusting of the Kazakh Shield has been suggested to account for the thickened crust and uplift in the northern part of the Tianshan due to the interpreted Moho doublet (UM and LM) with an overlap distance of ~100 km[3]. The Moho

doublet is also observed in our CCP images, but it is mostly confined to the vicinity of the NL where the overlap distance is no more than ~50 km (Supplementary Fig. 11d), which has been confirmed by systemic tests of the CCP stacking (Supplementary Figs. 6, 8, and 9). Given the V_S model beneath the IKA, the shallow Moho signals (UM) at a depth of ~30 km introduced by Zhang et al.[3] are likely a misinterpretation of the lower boundary of the banded low-V_S anomalies (Supplementary Fig. 11e). Moreover, the weak seismic activity in the southern margin of the Kazakh Shield supports the idea that southward underthrusting of the Kazakh Shield was very limited (Supplementary Fig. 11).

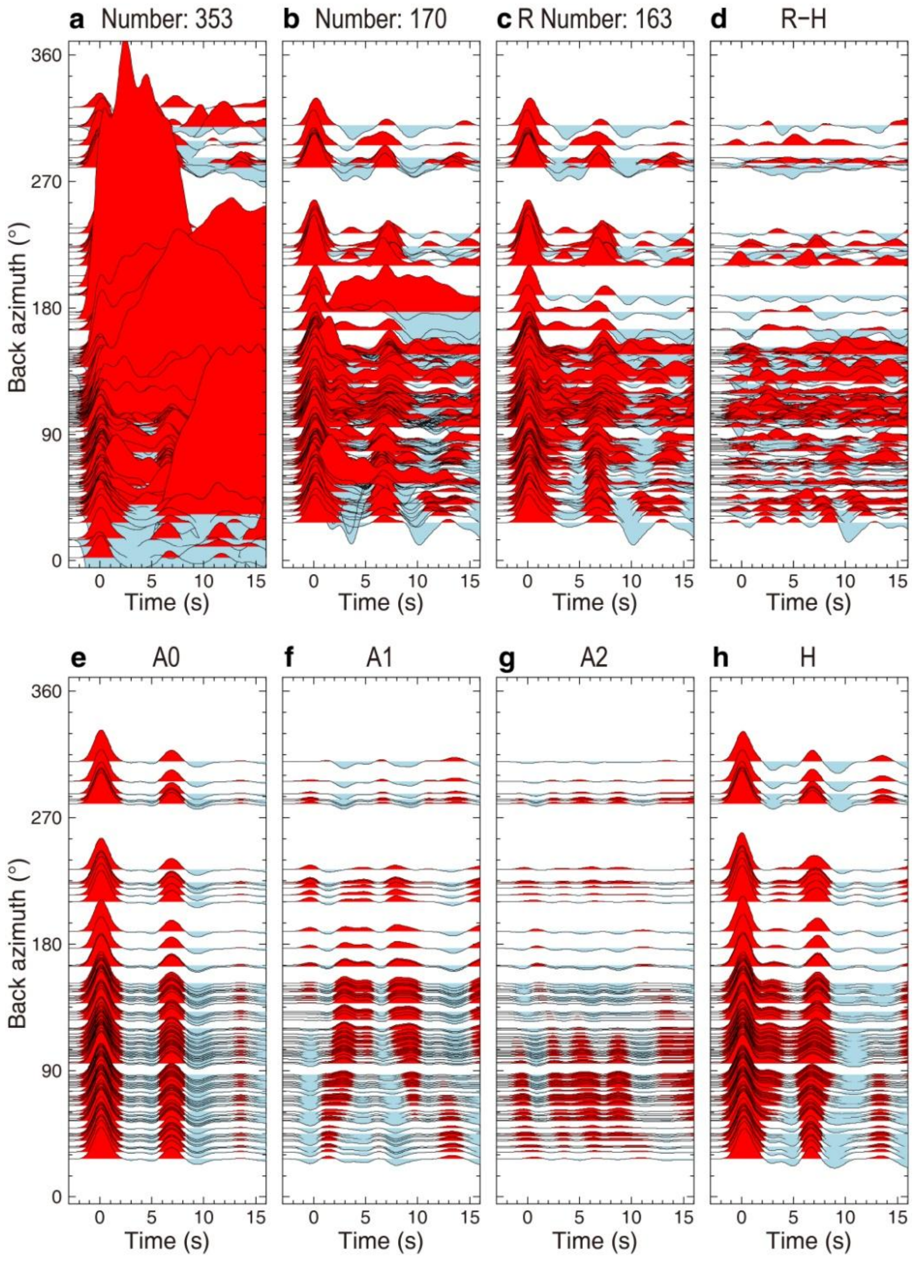

Supplementary Figure 1. Examples for the quality controls and harmonic analysis of P-wave receiver functions (RFs) with Gaussian coefficients 1.0. (a) All RFs observed at the station GOLB are plotted versus back-azimuth. **(b)** The residual RFs observed at the station GOLB after quality controls of amplitude and radial-component recovery. **(c)** The final RF dataset of the station GOLB after all quality controls, including the cross-correlation with bin-averaged RFs. **(d)** The difference between raw (R) and harmonic (H) RFs, where the harmonic RFs **(h)** are the sum of three harmonic components Ai (i = 0, 1, 2) **(e-g)** estimated from the reliable RFs dataset of the station GOLB.

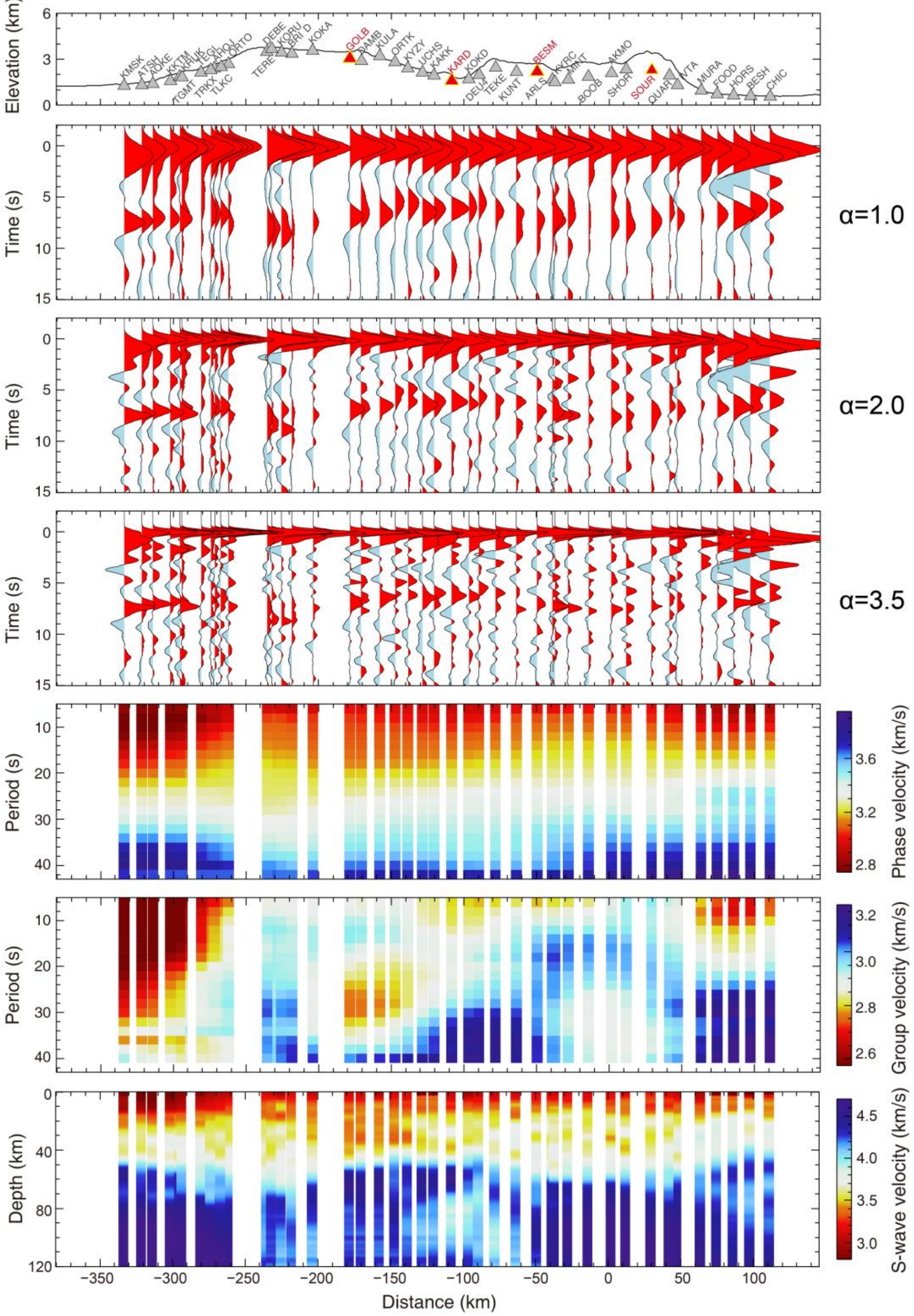

Supplementary Figure 2. Average RFs, interpolated Rayleigh wave dispersions (DPs), and inverted 1-D S-wave velocity (V_S) model at each station. (a) Station distribution (triangles with labels) with the topography along the profile. **(b–d)** The average RFs at each station along the profile with the Gaussian coefficients of 1.0, 2.0, and 3.5. **(e–f)** The interpolated DPs at each

station along the profile. **(g)** 1-D V_S model at each station obtained from the joint inversion in this study.

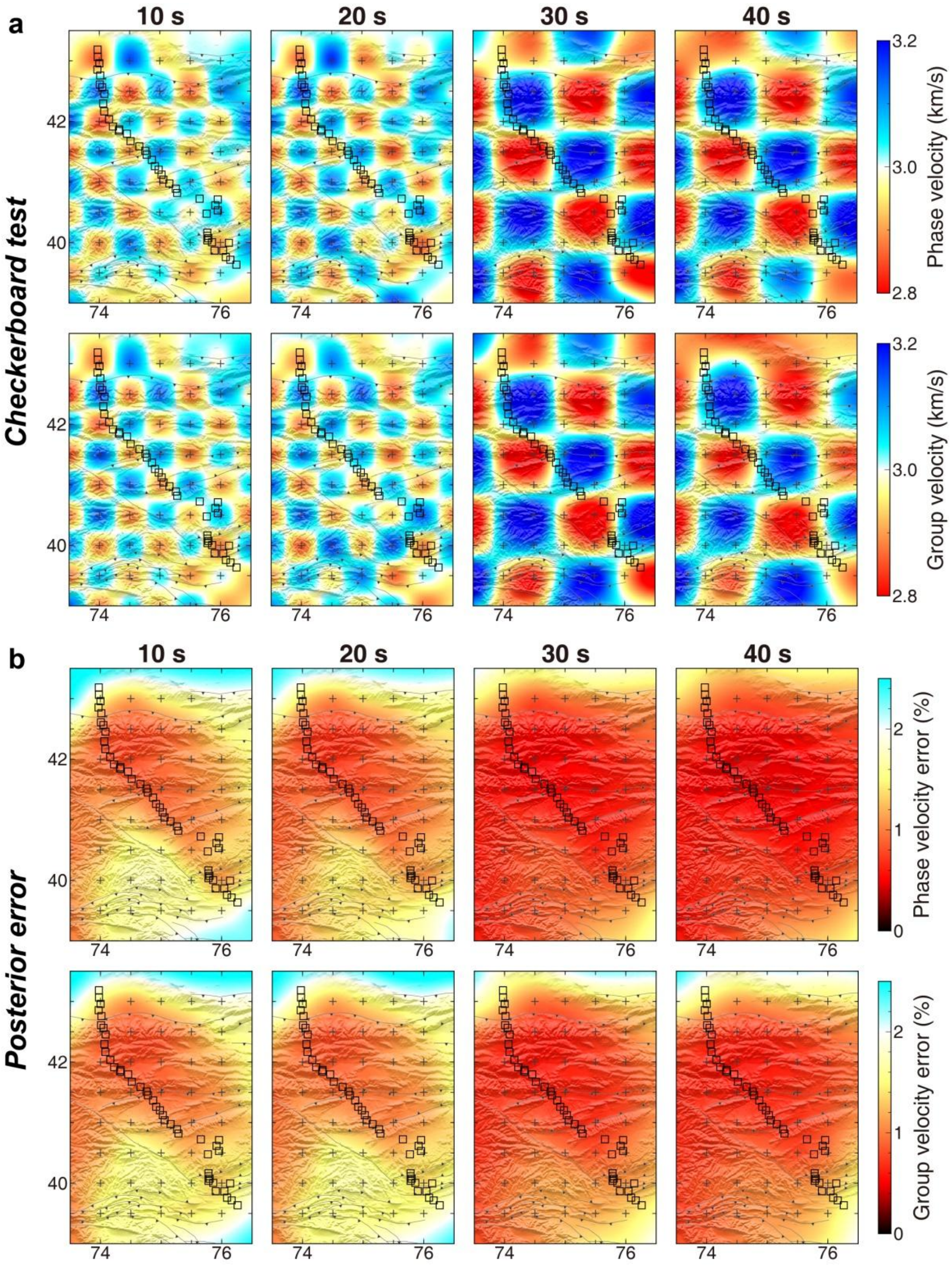

Supplementary Figure 3. Checkerboard tests and posterior errors of group and phase velocity DPs. (a) Checkerboard test of group and phase DPs for anomalies with the horizontal size of 0.5 ° × 0.5 ° for 10-s and 20-s periods, and 1.0 ° × 1.0 ° for 30-s and 40-s periods. **(b)** Posterior errors of group and phase DPs at periods of 10 s, 20 s, 30 s, and 40 s. Black crosses indicate grid nodes. Stations used in this study are shown as squares.

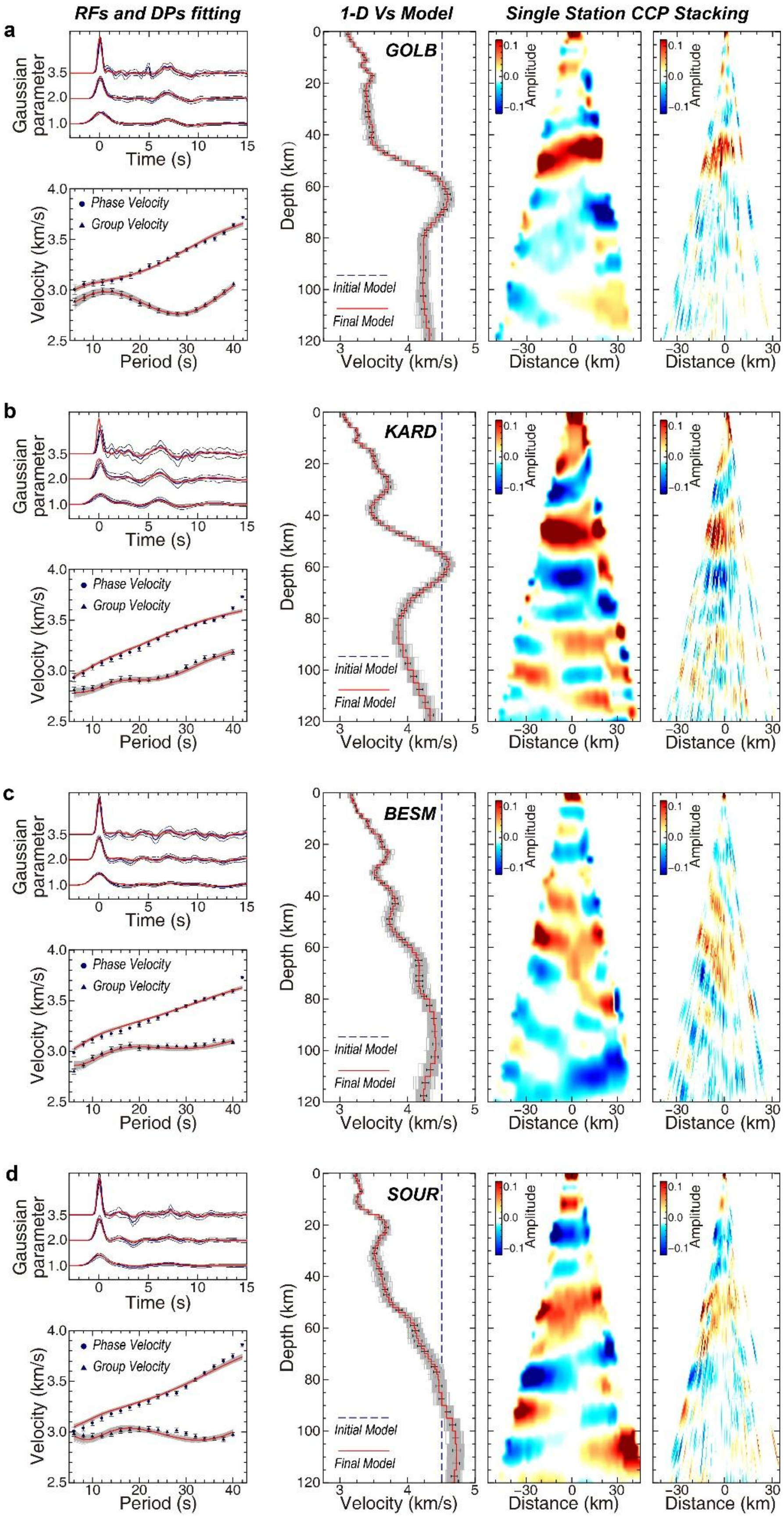
RFs and DPs fitting
1-D Vs Model
Single Station CCP Stacking
a
b
c
d
GOLB
KARD
BESM
SOUR
Gaussian parameter
Time (s)
Phase Velocity
Group Velocity
Velocity (km/s)
Period (s)
Depth (km)
Initial Model
Final Model
Amplitude
Distance (km)

Supplementary Figure 4. Examples of the joint inversion of RFs and DPs. (a) The RFs and DPs fitting of the station GOLB after the joint inversion. Dark blue lines with marks respectively denote the average RFs and interpolated DPs, in which circles for phase velocity and triangles for group velocity. Synthetic RFs and DPs calculated from final and resampling tests' inversion results are shown as red and gray lines. The final and initial models of the joint inversion are plotted as a red solid line and a blue dashed line, respectively. Gray lines indicate the inversion results of 200 resampling tests, and the error bars represent the average model of 200 resampling tests with a standard deviation. The single station CCP stacking results are shown with 5-km width stacking bins and individual traces of the reliable RFs along their raypaths in the depth-domain. **(b–d)** Same as (a), but for the station KARD, BESM, and SOUR. The locations of these stations are indicated in Figs. 1b and 2b.

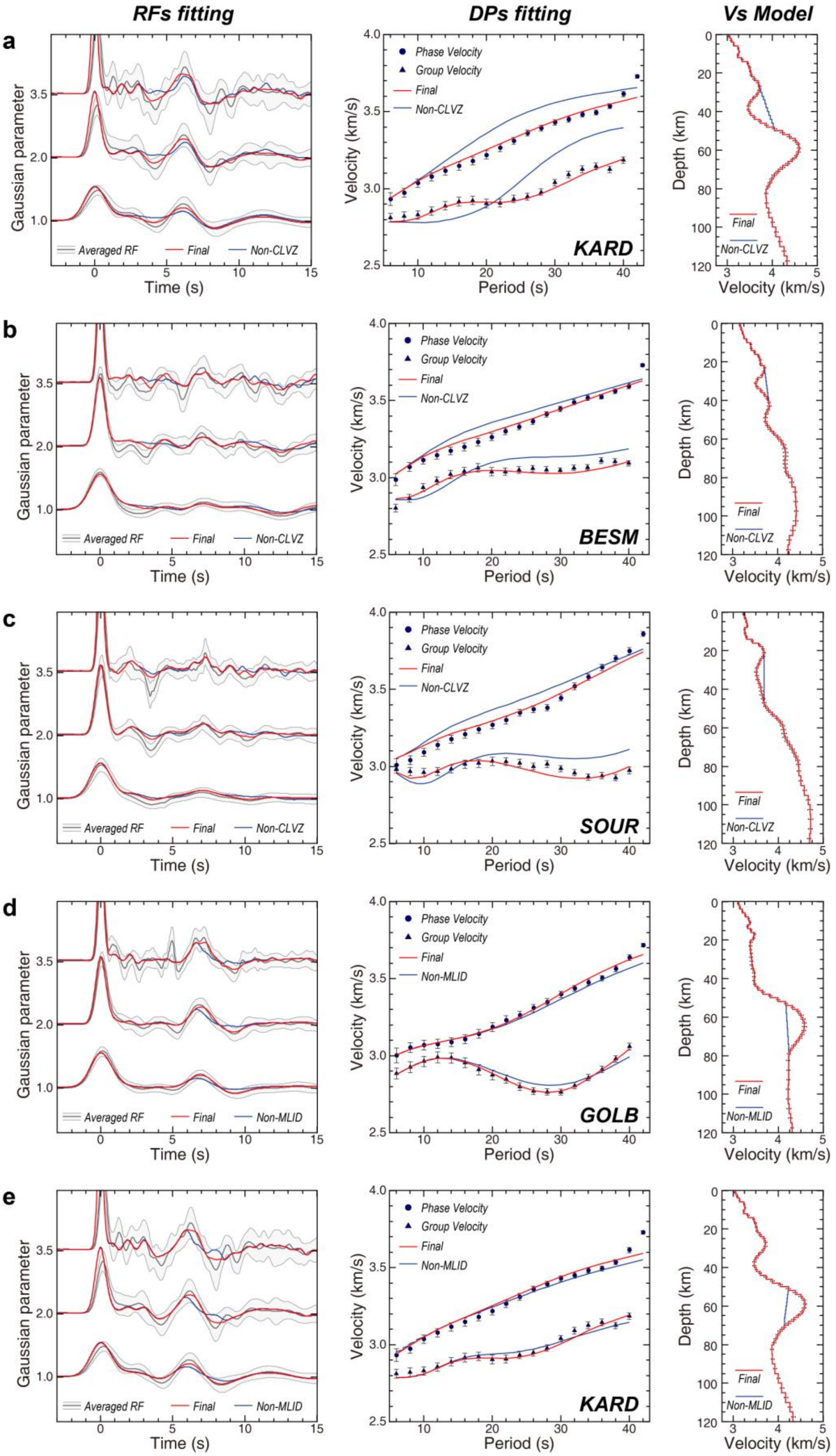
RFs fitting
DPs fitting
Vs Model
a
b
c
d
e
Gaussian parameter
Time (s)
Averaged RF
Final
Non-CLVZ
Non-MLID
Velocity (km/s)
Period (s)
Phase Velocity
Group Velocity
KARD
BESM
SOUR
GOLB
Depth (km)

Supplementary Figure 5. Robustness tests of crustal low-V_S anomalies and mantle lid structure. (a) Comparison of synthetic RFs and DPs of station KARD calculated from the final 1-D V_S model (red) and modified model without crustal low-V_S anomalies (green). Dark blue lines with marks respectively denote the average RFs and interpolated DPs, in which circles are for phase velocity and triangles for group velocity. Synthetic RFs and DPs calculated from final and modified models are shown as red and green lines. **(b–c)** Same as (a), but for stations BESM and SOUR. **(d–e)** Same as (a-c), but for the robustness test of the mantle lid structure beneath stations GOLB and KARD. The locations of these stations are indicated in Figs. 1b and 2b.

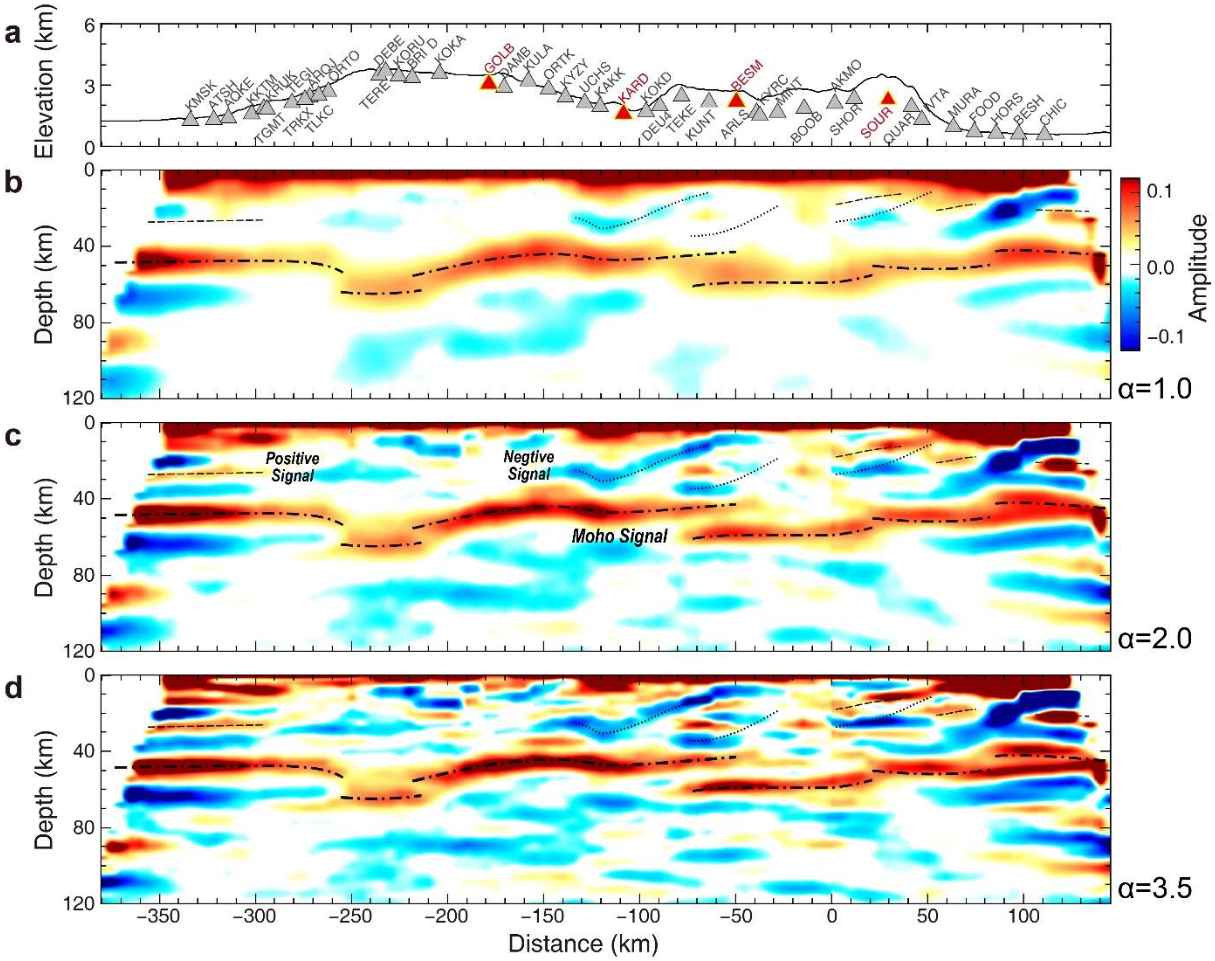

Supplementary Figure 6. Synthetic tests for effects of shallow structures in the CCP stacking. (a) Station distribution (triangles with labels) with the topography along the profile. **(b–d)** The CCP stacking results using the *Pms* phase of the reliable RFs with the Gaussian coefficient 1.0, 2.0, and 3.5. Black dashed lines represent the interpreted Moho and intracrustal positive/negative signals.

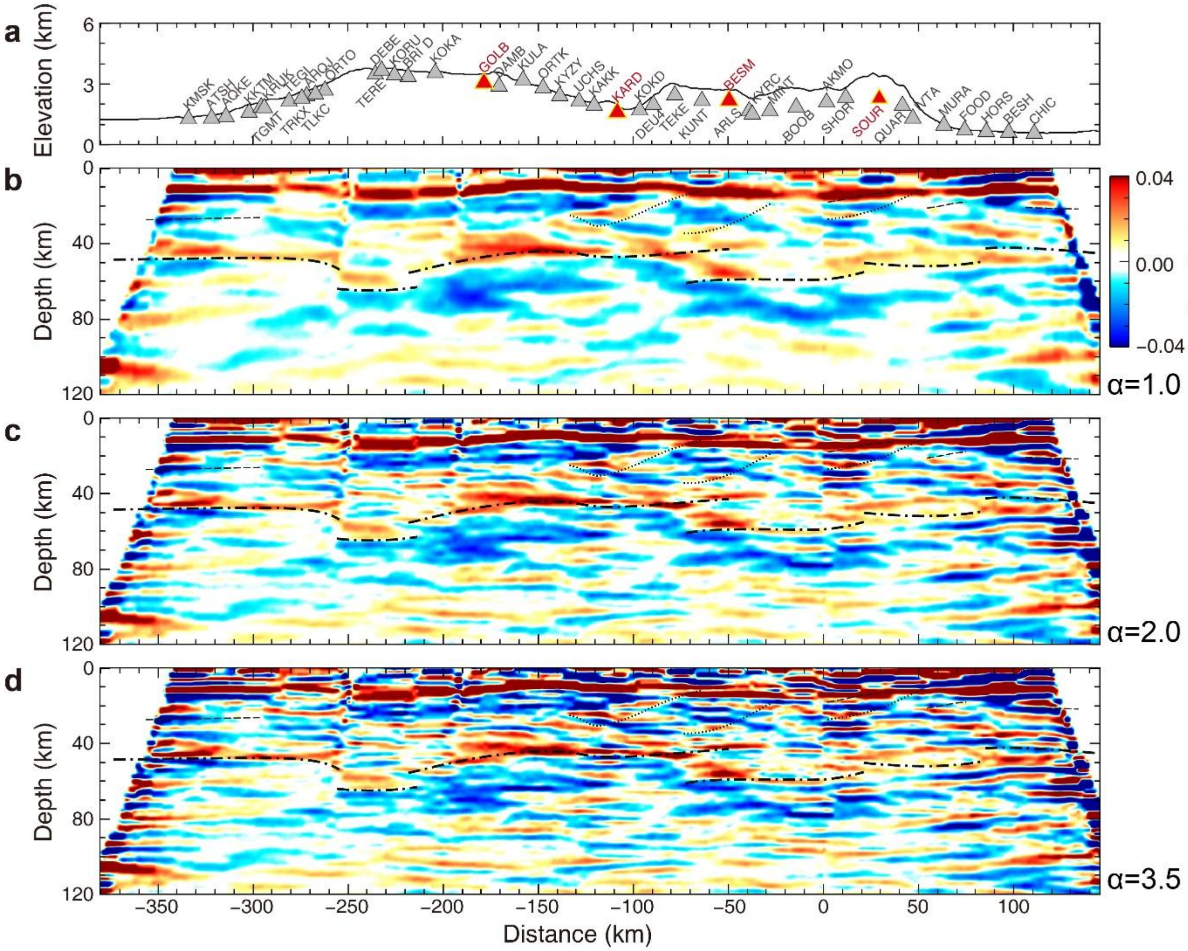

Supplementary Figure 7. CCP stacking results using the *PpPs* phase of the reliable RFs. (a) Station distribution (triangles with labels) with the topography along the profile. **(b–d)** The CCP stacking results using the *PpPs* phase of the reliable RFs with the Gaussian coefficient 1.0, 2.0, and 3.5. Black dashed lines represent the interpreted Moho and intracrustal positive/negative signals shown in Supplementary Fig. 6.

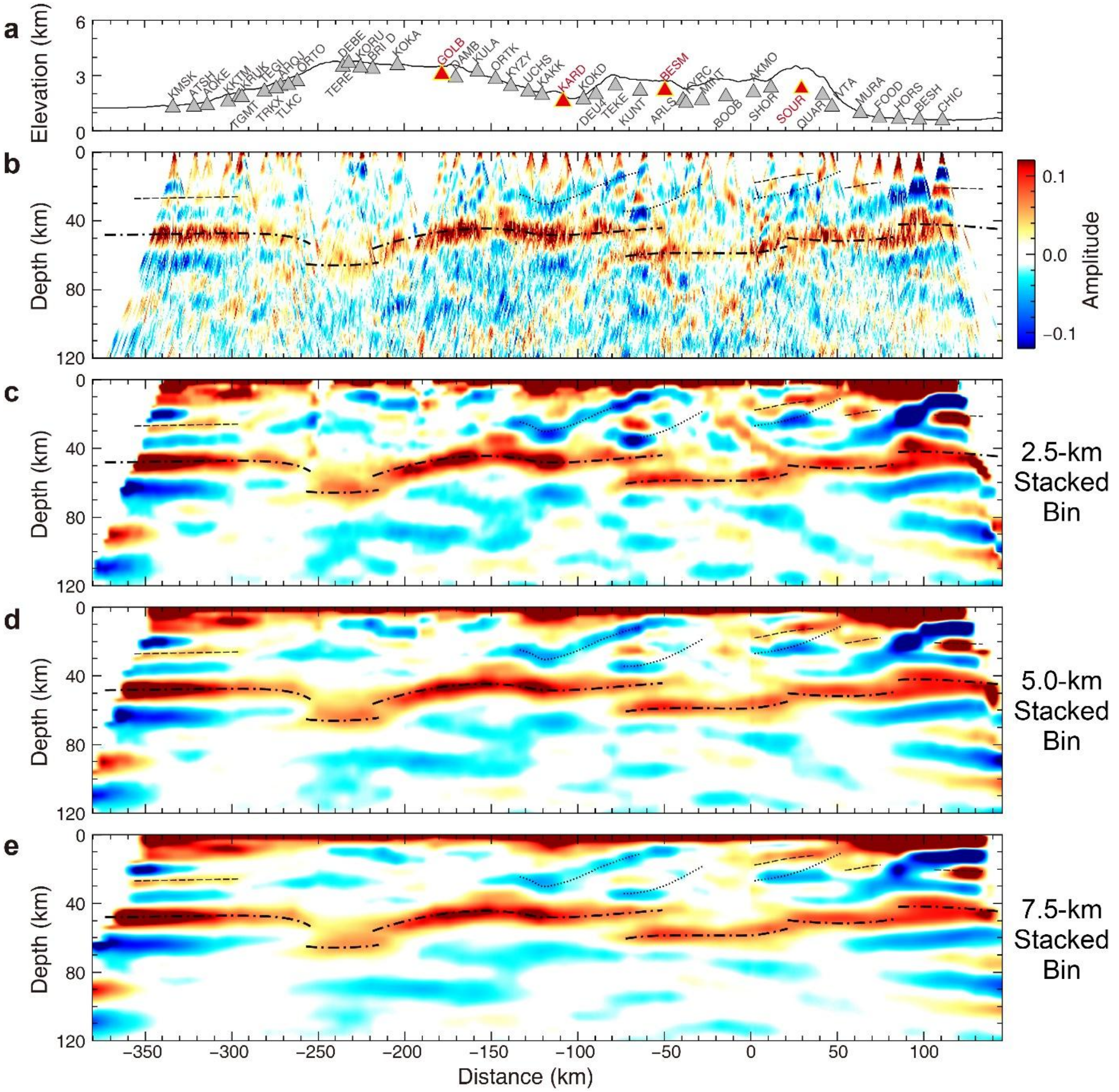

Supplementary Figure 8. Robustness tests of CCP stacking with different smoothing schemes. (a) Station distribution (triangles with labels) with the topography along the profile. **(b)** Individual traces of the reliable RFs plotted along their raypaths in the depth-domain. **(c–e)** The CCP stacking results using the *Ps* phase of the reliable RFs with different smoothing schemes. The width of the stacking bin parallel to the profile is set to 2.5 km, 5 km, and 7.5 km in the tests. The 5-km width stacking bin is used for constructing the CCP image in Fig. 2c. Black dashed lines represent the interpreted Moho and intracrustal positive/negative signals.

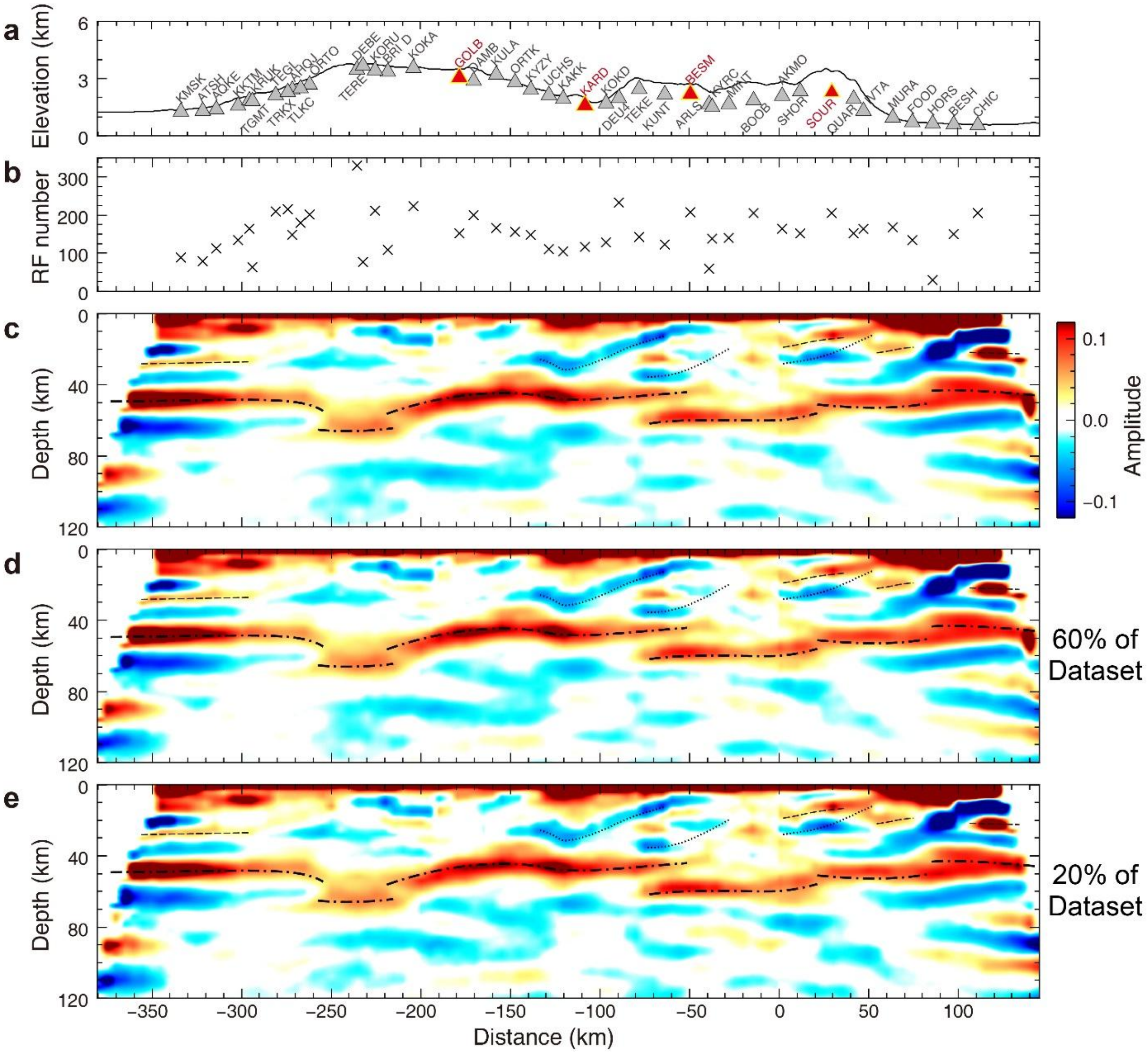

Supplementary Figure 9. Robustness tests of CCP stacking with different data amount. (a) Stations (triangles with labels) are plotted with the topography along the profile. **(b)** Numbers of the reliable RFs at each station. **(c–e)** The CCP stacking results using the *Ps* phase with different amount of the reliable RFs. 60% and 20% reliable RFs recorded at each station are extracted to construct limited datasets. Black dashed lines represent the interpreted Moho and intracrustal positive/negative signals.

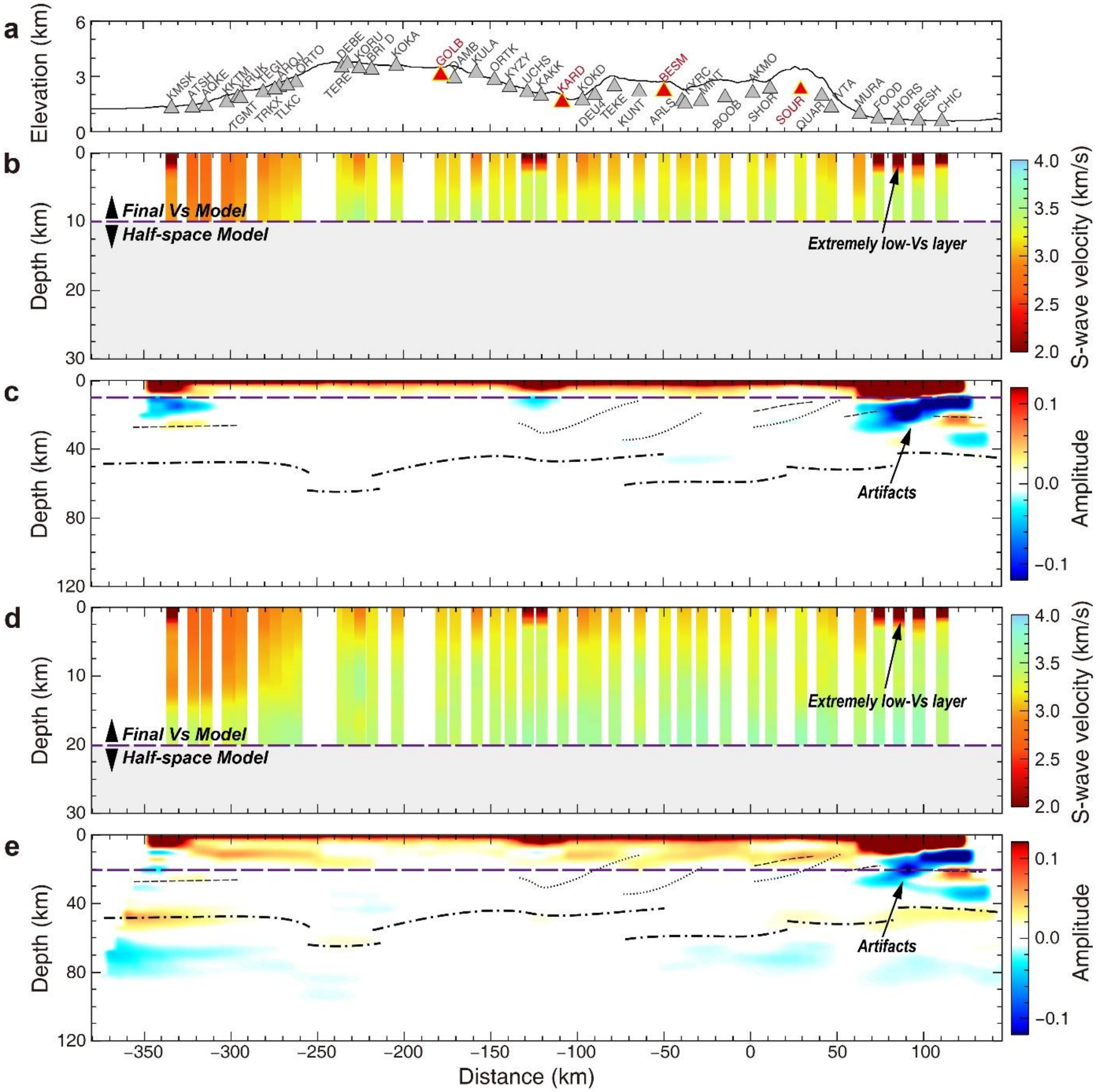

Supplementary Figure 10. Synthetic tests of shallow structures effects in the CCP stacking. **(a)** Stations (triangles with labels) are plotted with the topography along the profile. **(b)** The 1-D V_S model used in the synthetic test at each station, which is constructed by overlaying the final 1-D V_S model at depths shallower than 10 km on a homogeneous half-space model. **(c)** CCP stacked synthetic RFs for the models in (b) processed in the same way as for the real dataset. Significant artifact signals as strong negative *Ps* phase emerge in the north and south ends of the cross-section. **(d–e)** Same as (b–c), but for models shallower than 20 km overlaying a homogeneous half-space model. Black dashed lines represent the interpreted Moho and intracrustal positive/negative signals.

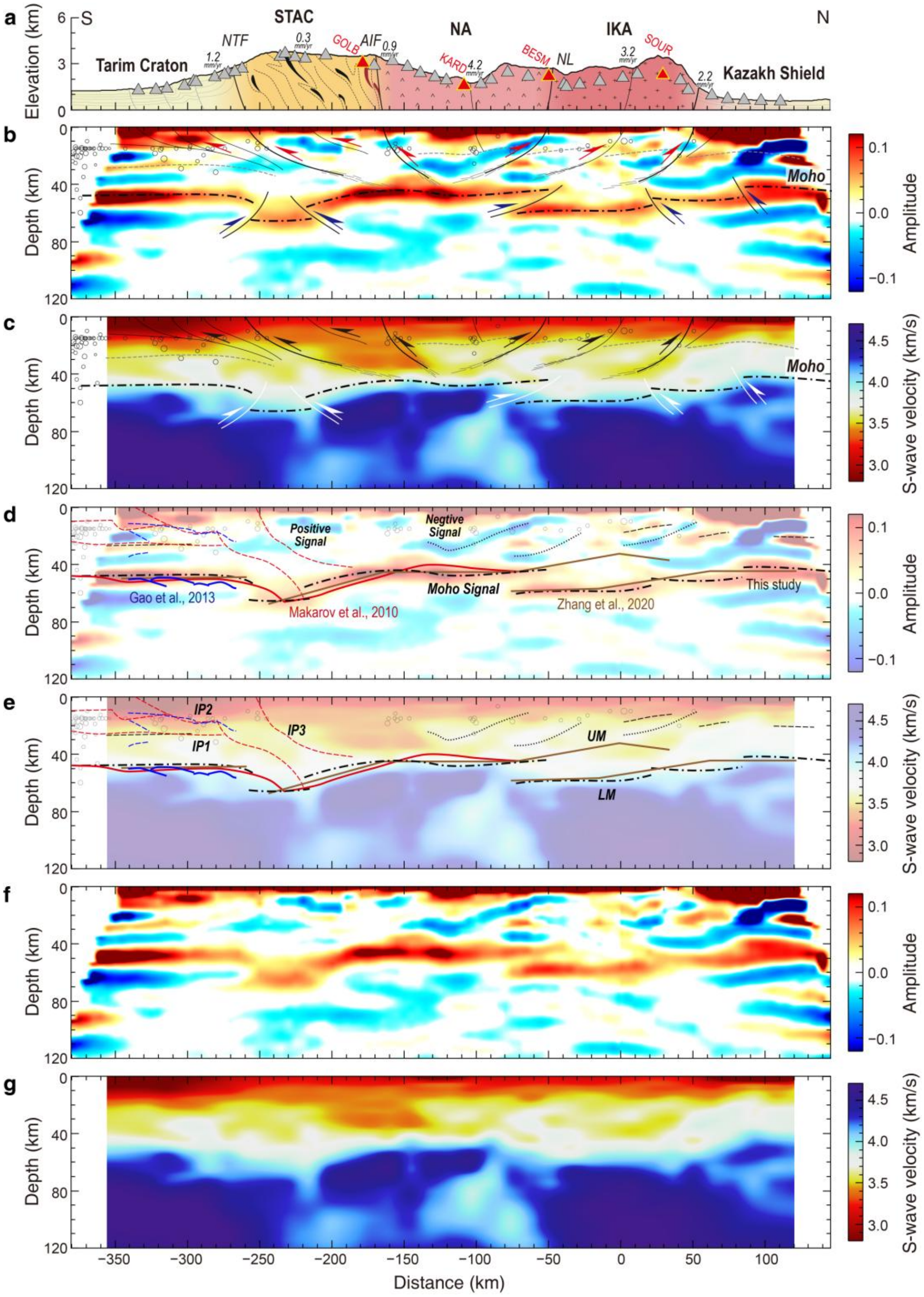

Supplementary Figure 11. Comparison of our result with previous studies. (a) Schematic geological cross-section across the Tianshan showing major tectonic units and the slip rates of main faults. Triangles represent stations used in this study, and red ones with marks indicate four stations chosen as examples showing the joint inversion in Supplementary Figs. 4 and 5. AIF:

Atbashy-Inylchek Fault, IKA: Issyk Kul Arc, NA: Naryn Arc, NL: Nikolaev Line, NTF: North Tarim Fault, KTB: Kepingtag Thrust Belt, STAC: Southern Tianshan Accretionary Complex. **(b–c)** The CCP stacking results with the Gaussian coefficient of 2.0 and the V_S model from joint inversion obtained in this study. These are plotted with interpreted faults (solid lines), Conrad (dashed thin lines) and Moho (dashed thick lines) discontinuities. Gray circles denote the earthquakes located within a 50-km width corridor centered by the cross-section[4]. **(d–e)** Crustal geometric and Vs structures marked interpretive signatures given by this and some previous studies along the same profile. The black solid and dashed lines mark the interpreted Moho and intracrustal positive/negative signals extracted from this study. Red, brown, and blue lines represent previous results from Gao et al.[1], Makarov et al.[2], and Zhang et al.[3], respectively. **(f–g)** Uninterpreted CCP stacking results and the V_S model same as shown in (a) and (b).

Supplementary References

1. Gao, R. *et al.* Fine crustal structure beneath the junction of the southwest Tian Shan and Tarim Basin, NW China. *Lithosphere* **5**, 382–392 (2013).

2. Makarov, V. I. *et al.* Underthrusting of Tarim beneath the Tien Shan and deep structure of their junction zone: Main results of seismic experiment along MANAS Profile Kashgar-Song-Köl. *Geotectonics* **44**, 102–126 (2010).

3. Zhang, B. F., Bao, X. W. & Xu, Y. X. Distinct orogenic processes in the South- and North-Central Tien Shan from receiver functions. *Geophys. Res. Lett.* **47**, e2019GL086941 (2020).

4. Engdahl, E. R. *et al.* ISC-EHB 1964–2016, an improved data set for studies of Earth structure and global seismicity. *Earth Space Sci.* **7**, e2019EA000897 (2020).
